# Determinants of caregiver burden in advanced Parkinson’s disease: a multidimensional and sex-sensitive perspective

**DOI:** 10.3389/fnagi.2026.1805627

**Published:** 2026-04-30

**Authors:** Laura Culicetto, Lilla Bonanno, Giulia Monea, Giulia Marafioti, Fabio Mauro Giambò, Giuseppe Di Lorenzo, Carmelo Mario Vicario, Mohammad Ali Salehinejad, Angelo Quartarone, Silvia Marino, Viviana Lo Buono

**Affiliations:** 1 IRCCS Centro Neurolesi “Bonino-Pulejo”, Messina, Italy; 2 Dipartimento di Medicina Clinica e Sperimentale, Università degli Studi di Messina, Messina, Italy; 3 Dipartimento di Scienze Cognitive, Psicologiche, Pedagogiche e Degli Studi Culturali, Università degli Studi di Messina, Messina, Italy; 4 School of Cognitive Sciences, Institute for Research in Fundamental Sciences (IPM), Tehran, Iran; 5 Department of Psychology and Neurosciences, Leibniz Research Centre for Working Environment and Human Factors, Dortmund, Germany; 6 Department of Child and Adolescent Psychiatry, Psychosomatics, and Psychotherapy, Medical Faculty, RWTH Aachen University, Aachen, Germany

**Keywords:** advanced stage, caregiver burden, Parkinson, rehabilitation, sex-differences

## Abstract

**Background:**

Parkinson’s disease (PD) is a progressive neurodegenerative disorder whose advanced stages (Hoehn & Yahr IV–V) place significant demands on informal caregivers, leading to multidimensional burden. Although functional and cognitive decline contribute to caregiver strain, the influence of sex and sex differences remains poorly understood.

**Objectives:**

This study examined the relationship between patients’ clinical characteristics and caregiver burden, and explored sex-based differences to identify sex-sensitive patterns.

**Methods:**

Thirty-eight individuals with advanced PD and their primary caregivers were assessed. Patients underwent clinical and neuropsychological evaluation using the Mini Mental State Examination (MMSE), Hamilton Rating Scales for Anxiety and Depression, Pittsburgh Sleep Quality Index (PSQI), Parkinson’s Disease Questionnaire (PDQ-39), and Activities of Daily Living (ADL/IADL) scales. Caregiver burden was measured with the Caregiver Burden Inventory (CBI). Associations were tested using Spearman’s rank correlations with false discovery rate correction, and analyses were stratified by patient sex.

**Results:**

Reduced patient autonomy was strongly associated with higher caregiver burden across emotional, physical, and time-dependent domains. Poorer quality of life, particularly mobility and bodily discomfort, further predicted caregiver strain. Female patients showed higher anxiety, but these symptoms were less related to caregiver burden. In contrast, functional and neuropsychiatric decline in male patients was more strongly linked to caregiver burden. Cognitive impairment was associated with increased caregiving time and demands. Female caregivers reported stronger links between patient dependence and burden.

**Conclusion:**

Functional decline, cognitive impairment, and reduced QoL in advanced PD significantly increase caregiver burden, with sex influencing these relationships. Sex-sensitive, multidisciplinary interventions are needed.

## 1 Introduction

Parkinson’s disease (PD) is a progressive neurodegenerative disorder that affects approximately 1–2% of individuals over 65 years of age, making it the second most prevalent neurodegenerative disease globally (Poewe et al., 2017). Characterized by the gradual loss of dopaminergic neurons in the substantia nigra, PD leads to motor symptoms such as bradykinesia, rigidity, resting tremor, and postural instability (Kalia and Lang, 2015). However, the disease extends beyond motor dysfunction, encompassing a broad spectrum of non-motor symptoms including depression, anxiety, apathy, alexithymia, sleep disturbances, cognitive decline, psychosis, and autonomic dysfunction (Chaudhuri et al., 2006; Moustafa et al., 2016; Culicetto et al., 2024, 2025).

As the disease progresses into the advanced stages, particularly Hoehn and Yahr (H&Y) stages IV and V, patients experience a substantial decline in both physical and cognitive functioning, severely compromising their autonomy and quality of life (QoL) (Hoehn and Yahr, 1967; Liu et al., 2018). This progressive loss of independence often necessitates continuous, intensive support from informal caregivers, most often spouses or close family members. These caregivers not only manage activities of daily living (ADLs) such as feeding, dressing, and hygiene, but also oversee instrumental activities of daily living (IADLs) including medication administration, financial planning, and communication with healthcare providers (Depp and Jeste, 2006; Millán-Calenti et al., 2010).

The term “caregiver burden” refers to the multidimensional strain experienced by individuals who provide care, encompassing emotional, physical, social, financial, and psychological domains (Zarit et al., 1986). This burden is particularly pronounced in PD due to its chronic, incurable nature and the complexity of its symptomatology. In many cases, the caregiver becomes a “hidden patient” within the clinical triad, experiencing significant emotional distress, social isolation, and even physical health deterioration (Mosley et al., 2017). Long-term caregiving also imposes economic costs, despite the societal benefit of delaying institutionalization and reducing reliance on formal healthcare systems (Arno et al., 1999).

Research shows that reduced patient functionality, especially in ADLs and IADLs, is a strong predictor of caregiver burden (Genç et al., 2019; Rosqvist et al., 2022). Functional impairment limits patients’ ability to participate in everyday life and often reflects broader neuropsychiatric deterioration, including executive dysfunction (Chan et al., 2008; Hunter and Kearney, 2018; Emre, 2007). As a result, caregivers assume increasing responsibility, often exceeding 20 h per week (Rosqvist et al., 2022). These caregiving duties may be further exacerbated by treatment-related factors, including adverse effects of dopaminergic therapies (such as impulse control disorders or psychotic symptoms), the complexity of medication scheduling, and device-related care needs such as postoperative management or maintenance of infusion and stimulation-based therapies, all of which may destabilize care routines and add to emotional strain (Chaudhuri et al., 2006).

Sex differences in PD have been increasingly recognized across both motor and non-motor domains. Women with PD more frequently report anxiety, depression, pain, sleep disturbances, and poorer emotional QoL, whereas men may more often show profiles marked by cognitive decline, gait/postural impairment, or other manifestations with different implications for daily care needs (Cattaneo and Pagonabarraga, 2025). At the same time, growing evidence suggests that caregiver burden in PD may also vary according to the sex of both patients and caregivers. In a large cohort from the Parkinson’s Outcomes Project, men with PD were more likely than women to have an informal caregiver, and caregivers of male patients reported greater strain (Dahodwala et al., 2018). More recently, Horn et al. (2025) reported complementary findings showing that women caregivers, who more often cared for men with PD, experienced higher strain, had less time available for other family members, and more frequently cared for patients with greater disability and cognitive decline. Within this framework, the present study adds to the literature by focusing specifically on advanced PD, examining a different cultural and healthcare context, and exploring sex-sensitive patterns primarily according to patient sex.

Despite growing recognition of the caregiving crisis in PD, there is limited research focusing on the specific challenges faced by caregivers of patients in the late stages of the disease. Moreover, existing studies often fail to account for sex-based differences that may influence both patient presentation and caregiver experience. For instance, female patients may report higher levels of emotional distress, while male patients may present with more externally disruptive behaviors, each pattern eliciting different caregiving responses (Genç et al., 2019; Rosqvist et al., 2022).

The present study aims to investigate the complex interplay between patient-related clinical variables and caregiver burden in the context of advanced PD. In addition, the research explores whether and how these associations differ according to the sex of both patients and caregivers, with the aim of identifying sex-specific patterns that may inform the design of more tailored support strategies. By examining these dynamics within a real-world, multidisciplinary care setting, the study seeks to generate insights that can enhance the development of personalized, sex-sensitive interventions to sustain caregivers’ well-being and improve the overall management of late-stage PD.

## 2 Methods

### 2.1 Study participants and design

This exploratory study involved thirty-eight Italian individuals of Caucasian ethnicity with idiopathic PD (70.66 ± 7.63 years; 15 females) diagnosed in routine clinical practice by a neurologist specialized in movement disorders according to the criteria established by the Movement Disorder Society (MDS) (Postuma et al., 2015), along with their primary caregivers. Patients were recruited from the Day Hospital of the IRCCS Centro Neurolesi “Bonino-Pulejo” in Messina between January 2019 and March 2020. Eligible patients and their primary caregivers, identified as the persons mainly involved in the patients’ daily care, were invited by the clinical psychologist to participate in the study. Thirty-eight caregivers (62.76 ± 12.81 years; 23 females) were eventually interviewed. Eligibility criteria for caregivers included being at least 18 years of age, having no significant cognitive impairments, and being free from serious medical conditions.

This study was approved by the Ethical and Research Committee of IRCCS Centro Neurolesi Bonino Pulejo, Messina, Italy (ID: 08/2018) and conducted in accordance with the principles of the 1964 Declaration of Helsinki.

Patients meeting inclusion criteria and with a primary caregiver willing to participate were enrolled. Both the patient and their caregiver provided informed consent for participation and were assessed individually. Confidentiality of data and personal information was assured to the participants.

A single, comprehensive assessment was performed for each patient-caregiver dyad by a multidisciplinary team composed of a clinical psychologist and a neurologist specialized in movement disorders. Demographic and clinical data were collected for both patients and caregivers, including age, sex, and educational level. For patients, additional information regarding disease duration and pharmacological treatment was also recorded.

### 2.2 Inclusion criteria for patients with Parkinson’s disease (PD)

Inclusion criteria consisted of a diagnosis of late-stage idiopathic PD, defined as a disease duration of at least 10 years and a H&Y stage of IV or V, or the presence of marked disability, indicated by a Schwab and England scale score of 50% or less in the ‘on’ medication state. Additional inclusion criteria were age between 40 and 90 years and a minimum of 5 years of formal education (either primary or secondary school level). Subjects with atypical parkinsonian syndromes, including progressive supranuclear palsy (PSP), corticobasal degeneration (CBD), dementia with Lewy bodies (DLB), and multiple system atrophy (MSA), were excluded from the study. Subjects with potentially reversible forms of parkinsonism, such as normal pressure hydrocephalus or drug-induced parkinsonism, were also excluded.

### 2.3 Procedure and clinical evaluation

All PD subjects underwent a standardized neurological assessment that included the H&Y staging for disease severity. In addition, a comprehensive battery of neuropsychological and functional assessments was administered to evaluate cognitive functioning, mood, QoL, sleep, and autonomy in daily activities.

#### 2.3.1 Cognitive function

- Mini-Mental State Examination (MMSE): This widely used screening tool evaluates global cognitive functioning, including orientation, memory, attention, language, and visuospatial skills. Scores range from 0 to 30, with scores above 24 considered indicative of normal cognitive performance (Folstein et al., 1975).

#### 2.3.2 Mood and psychological symptoms

- Hamilton Rating Scale for Depression (HAM-D or HRS-D): A clinician-rated scale used to assess the severity of depressive symptoms. Scores range from 0 to 52, with a cut-off of ≥17 indicating clinically significant depression (Hamilton, 1960).
- Hamilton Rating Scale for Anxiety (HAM-A or HRS-A): A clinician-rated tool assessing the intensity of anxiety symptoms. The scale ranges from 0 to 56, with scores ≥18 reflecting clinically significant anxiety (Hamilton, 1959).

#### 2.3.3 Sleep quality

- Pittsburgh Sleep Quality Index (PSQI): A self-report instrument measuring sleep quality and disturbances over a one-month interval. It includes seven components, such as sleep latency, duration, and disturbances, producing a global score (higher scores indicate poorer sleep quality) (Buysse et al., 1989).

#### 2.3.4 Health-related quality of life

- Parkinson’s Disease Questionnaire (PDQ-39): A disease-specific, self-administered questionnaire that assesses the impact of PD on QoLacross eight dimensions: mobility, activities of daily living, emotional well-being, stigma, social support, cognitions, communication, and bodily discomfort. Total scores range from 0 to 156, with higher scores indicating greater impairment. There is no fixed clinical cut-off; interpretation is based on overall and domain-specific distributions (Jenkinson et al., 1997).

#### 2.3.5 Functional autonomy and activities of daily living

- The Activities of Daily Living (ADL) scale, developed by Katz (1963), is a standardized tool used to assess an individual’s functional independence in essential self-care tasks. It is widely applied in clinical settings such as geriatrics, neurology, and rehabilitation to determine the level of assistance a person requires in daily life. The scale evaluates six basic activities: bathing, dressing, toileting, transferring, continence, and feeding. Each activity is scored as 1 if performed independently or 0 if assistance is needed, for a total score ranging from 0 (complete dependence) to 6 (full independence). A lower total score reflects greater disability and increased need for support. The ADL scale provides a quick and reliable overview of a person’s self-care capacity and is especially useful in conditions like PD, stroke, or dementia.
- The Instrumental Activities of Daily Living (IADL) score was assessed using the criteria established by Lawton and Brody (Graf, 2009). This questionnaire includes eight items that evaluate an individual’s ability to independently perform tasks such as using the telephone, shopping, food preparation, housekeeping, laundry, transportation, medication management, and financial handling. The IADL score extends the assessment of basic ADL by capturing a broader range of daily functions and the capacity to live independently within the community (Spector et al., 1987). A total score was calculated by summing the individual item scores, with a maximum possible score of 8. Participants who achieved the full score were classified as having ‘no limitations,’ whereas those with a score of less than 8, indicating at least one item rated as requiring assistance, were categorized as having limitations.

#### 2.3.6 Assessment of caregiver burden

Caregivers were asked to complete the Caregiver Burden Inventory (CBI), a multidimensional, self-report questionnaire originally developed to assess the burden experienced by caregivers of individuals with Alzheimer’s disease (Novak and Guest, 1989). The CBI consists of 24 items and is designed to capture five distinct dimensions of caregiver burden: time-dependence, developmental burden, physical burden, social burden, and emotional burden. Each item is rated on a 5-point Likert scale, with higher total scores indicating a greater overall burden. Although the CBI has not been specifically validated in PD populations, prior research has demonstrated its sensitivity to changes following psychosocial interventions in caregivers of PD subjects(Mosley et al., 2017). This supports its applicability for assessing perceived burden in this context. The multidimensional nature of the CBI allows for a nuanced evaluation of the different stressors caregivers may face, which is particularly relevant in advanced stages of PD, where caregiving often involves both physical assistance and emotional strain.

### 2.4 Statistical analysis

A descriptive analysis was performed for demographic and clinical variables. Continuous variables were reported as mean ± standard deviation when the Shapiro–Wilk test did not reject normality (*p* ≥ 0.05), and as median with interquartile range when the distribution was non-normal, categorical variables are presented as absolute counts and percentages. Because several scales showed non-normal distributions, associations between patients’ clinical scores and the six caregiver-burden dimensions were examined with Spearman’s rank correlation, and *p*-values were adjusted for multiple testing by the Benjamini–Hochberg false-discovery rate (FDR), considering results significant at FDR-adjusted *p* < 0.05. Subsequently, the patient cohort was then stratified by sex, for each continuous variable the comparison between males and females was performed using the unpaired Student’s t-test, when normality assumptions were met, or with the U Mann–Whitney, when violated, reporting Cohen’s d for parametric tests and effect-size for non-parametric tests. The same approach was applied to compare male versus female caregivers. Finally, Spearman correlations between patient scores and caregiver-burden indices were recomputed within each subgroup (male patients, female patients, male caregivers, female caregivers) to explore sex-specific patterns. All statistical analyses were conducted using the open-source software R 4.2.2. A significance threshold of *p* < 0.05 was adopted, with 95% confidence intervals and a 5% alpha error.

## 3 Results

### 3.1 Baseline demographic and clinical characteristics

Baseline demographic and clinical characteristics of the patient cohort are reported in Table 1. The sample included 38 individuals with advanced PD (mean age 70.66 ± 7.63 years; 15 females), with a long disease duration and advanced disease stage. No significant sex differences were observed in age, education, disease duration, H&Y or levodopa equivalent daily dose (all *p* > 0.05). However, significant sex-related differences emerged in several non-motor and QoL domains. Female patients showed higher levels of HAM-A, greater PDQ-39 emotional well-being, increased bodily discomfort, and more severe sleep disturbances compared with male patients (Table 1). The caregiver sample included 38 primary caregivers (mean age 62.76 ± 12.81 years; 23 females). Most caregivers were wife/husband (76.3%), followed by son/daughter (21.1%), with a small proportion of other relatives. In most cases, caregiving involved opposite-sex pairs (84.2%), with female caregivers predominantly assisting male patients (Table 2). No significant sex differences were observed in caregiver burden dimensions among caregivers (Table 2).

**Table 1:** Demographic and clinical characteristics of the patient, stratified by sex.

Variables	All	Male	Female	*p*	Effect size
N. subjects	38	23	15		
Age	70.66 ± 7.63	71.96 ± 7.85	68.67 ± 7.08	0.19	*d* = 0.43
Education	10.92 ± 4.77	11.17 ± 5.16	10.50 ± 4.22	0.69	*d* = 0.14
Disease duration	11.0 (10.0–12.0)	11.0 (10.0–12.5)	11.0 (10.0–12.0)	0.94	*r* = 0.01
H&Y	4.0 (4.0–4.0)	4.0 (4.0–4.0)	4.0 (4.0–4.0)	0.53	*r* = 0.10
LEED	829.84 ± 281.59	892.48 ± 304.67	733.80 ± 217.64	0.07	*d* = 0.58
IADL	4.50 (2.25–4.5)	4.0 (2.0–7.0)	5.0 (3.0–6.5)	0.72	*r* = 0.06
ADL	4.50 (2.0–4.5)	4.0 (2.0–5.0)	5.0 (3.0–5.5)	0.46	*r* = 0.12
HAM-D	18.32 ± 7.97	17.04 ± 7.80	20.27 ± 8.09	0.23	*d* = −0.41
HAM-A	20.24 ± 9.63	17.35 ± 7.56	24.67 ± 10.97	0.03*	*d* = −0.81
MMSE	23.53(20.89–23.53)	22.85 (17.43–27.69)	24.46 (22.87–26.74)	0.45	*r* = 0.13
PDQ SI	44.55 (37.98–54.17)	44.87 (18.27–52.56)	44.23 (42.95–58.01)	0.19	*r* = 0.21
PDQ mobility	66.25 (47.50–66.25)	67.50 (30.0–80.0)	65.0 (55.0–83.75)	0.33	*r* = 0.16
PDQ ADL	35.42 (29.17–37.5)	33.33 (20.83–87.5)	41.67 (25.0–58.3)	0.93	*r* = 0.02
PDQ emotional well being	43.74 ± 22.17	33.31 ± 16.03	59.72 ± 21.05	<0.001*	*d* = −1.46
PDQ stigma	28.13 (0–28.12)	12.50 (0–40.62)	37.50 (18.75–50.0)	0.12	*r* = 0.26
PDQ social support	0 (0–16.67)	0 (0–16.67)	8.33 (0–16.67)	0.45	*r* = 0.13
PDQ cognitions	31.25 (25.0–50.0)	25.0 (15.63–50.0)	34.38 (25.0–54.69)	0.24	*r* = 0.20
PDQ communication	33.33 (8.33–50.0)	33.33 (12.50–50.0)	41.67 (10.42–54.17)	0.76	*r* = 0.05
PDQ Bodily discomfort	58.33 (41.67–83.33)	50.0 (29.17–75.0)	75.0 (54.17–83.33)	0.02*	*r* = 0.38
Pittsburgh Sleep Quality Index	9.50 ± 3.69	8.52 ± 3.86	11.0 ± 2.93	0.03*	*d* = −0.70
Subjective sleep quality	1.50 (1.0–2.0)	1.0. (1.0–2.0)	2.0 (1.0–2.5)	0.05	*r* = 0.33
Sleep latency	2.0 (1.0–2.0)	1.0 (0.0–2.0)	2.0 (1.0–2.0)	0.29	*r* = 0.17
Sleep duration	2.0 (2.0–3.0)	2.0 (1.50–3.0)	2.0 (2.0–2.5)	0.97	*r* = 0.01
Habitual sleep efficiency	0.0 (0.0–0.0)	0.0 (0.0–0.0)	0.0 (0.0–0.0)	0.37	*r* = 0.15
Sleep disturbances	2.0 (1.25–2.0)	2.0 (1.0–2.0)	2.0 (2.0–3.0)	0.02*	*r* = 0.37
Use of sleep medication	0.0 (0.0–3.0)	0.0 (0.0–3.0)	1.0 (0.0–3.0)	0.42	*r* = 0.13
Daytime dysfunction	1.0 (0.25–2.0)	1.0 (0.0–2.0)	1.0 (1.0–2.0)	0.56	*r* = 0.10

Data are presented as mean ± standard deviation for normally distributed variables, and as median (interquartile range) for non-normally distributed variables. Between-group comparisons were performed using Student’s *t*-test or U Mann–Whitney test as appropriate. Effect sizes are reported as Cohen’s *d* for parametric comparisons and as *r* for non-parametric comparisons. *p < 0.05. Cohen’s *d* (*d*) represents the standardized mean difference between two groups and is used for normally distributed data. Effect sizes are reported as Cohen’s *d* for parametric comparisons and as *r* for non-parametric comparisons. Effect size *r* (*r*) represents the rank-biserial correlation and is used for non-normally distributed data.

**Table 2:** Demographic and clinical characteristics of the caregiver stratified by sex.

	All	Male	Female	*p*	Effect size
N. subjects	38	15	23		
Age	62.76 ± 12.81	71.96 ± 7.85	68.67 ± 7.08	0.89	*d* = −0.05
Education	10.92 ± 3.69	11.17 ± 5.16	10.50 ± 4.22	0.46	*d* = 0.12
Relationship to patient, *n* (%)					
Wife/Husband	29 (76.3%)	11 (73.3%)	18 (78.3%)		
Son/Daughter	8 (21.1%)	4 (26.7%)	4 (17.4%)		
Other	1 (2.6%)	0 (0%)	1 (4.3%)		
Sex of patient cared for, *n* (%)					
Male	23 (60.5%)	3 (20.0%)	20 (87.0%)		
Female	15 (39.5%)	12 (80.0%)	3 (13.0%)		
Caregiver-patient sex pairing, *n* (%)					
Opposite sex	32 (84.2%)	12 (80.0%)	20 (87.0%)		
Same sex	6 (15.8%)	3 (20.0%)	3 (13.0%)		
CBI	24.97 ± 14.75	22.27 ± 10.71	26.74 ± 16.87	0.32	*d* = −0.30
Assistance time	10.37 ± 5.68	10.33 ± 4.22	10.39 ± 6.55	0.97	*d* = −0.01
Perceived progression	6.11 ± 4.56	5.40 ± 3.72	6.57 ± 5.06	0.42	*d* = −0.25
Physical burden	4.0 (1.0–7.75)	3.0 (1.0–5.50)	4.0 (1.50–10.50)	0.32	*r* = 0.16
Social burden	1.0 (0.0–3.0)	1.0 (0.0–2.50)	2.0 (0.50–3.50)	0.21	*r* = 0.20
Emotional burden	1.0 (0.0–1.0)	1.0 (0.0–1.0)	1.0 (0.0–2.50)	0.69	*r* = 0.07

Data are presented as mean ± standard deviation for normally distributed variables and median (interquartile range) for non-normally distributed variables. Categorical variables are reported as counts and percentages. Between-group comparisons were performed using Student’s *t*-test or Mann–Whitney U test as appropriate. Cohen’s *d* (*d*) represents the standardized mean difference between two groups and is used for normally distributed data. Effect size *r* (*r*) represents the rank-biserial correlation and is used for non-normally distributed data.

### 3.2 Correlations between patients’ and caregivers’ measures

Several significant associations emerged between patients’ clinical status and caregiver-related variables. Lower functional autonomy, as assessed by both IADL and ADL, was strongly associated with higher caregiver burden, increased time dedicated to assistance, and higher perceived disease progression (all FDR-corrected *p* < 0.05; Table 3). Similarly, worse patient-reported QoL, particularly in the PDQ-39 mobility and ADL domains, was associated with increased caregiver burden and caregiving demands (Table 3). Bodily discomfort also showed positive associations with caregiver burden and time dedicated to assistance, although these did not survive correction for multiple comparisons. No significant associations were observed for emotional burden after FDR correction (Table 3).

**Table 3:** Spearman correlation coefficients between patient clinical variables and caregiver burden dimensions.

Patient variable	Caregiver outcome	Spearman’s ρ	*p*-value	FDR-adjusted *p*
IADL	CBI	−0.67	<0.001	**<0.001**
	Time dedicated to assistance	−0.59	<0.001	**<0.001**
	Perceived disease progression	−0.61	<0.001	**<0.001**
	Physical burden	−0.52	0.001	**0.01**
	Emotional burden	−0.32	0.05	0.33
ADL	Caregiver burden (CBI)	−0.64	<0.001	**<0.001**
	Time dedicated to assistance	−0.68	<0.001	**<0.001**
	Perceived disease progression	−0.57	<0.001	**<0.001**
	Physical burden	−0.41	0.01	0.10
	Emotional burden	−0.30	0.07	0.38
PDQ mobility	Caregiver burden (CBI)	0.53	0.001	**0.01**
	Time dedicated to assistance	0.45	0.004	**0.04**
	Perceived disease progression	0.49	0.002	**0.02**
	Social burden	0.31	0.06	0.36
PDQ ADL	Caregiver burden (CBI)	0.54	<0.001	**0.01**
	Time dedicated to assistance	0.51	0.001	**0.01**
	Perceived disease progression	0.48	0.002	**0.03**
	Physical burden	0.36	0.03	0.20
PDQ bodily discomfort	Caregiver burden (CBI)	0.36	0.03	0.20
	Time dedicated to assistance	0.40	0.02	0.13
	Perceived disease progression	0.35	0.03	0.22

*p*-values were adjusted for multiple comparisons using the Benjamini–Hochberg false discovery rate (FDR). Significant associations (FDR < 0.05) are highlighted in bold.

### 3.3 Sex differences in clinical and quality-of-life measures in patient group

After stratifying the cohort, a series of between-group comparisons were conducted to explore potential sex differences in clinical and quality-of-life variables (Table 1).

Female patients showed higher levels of anxiety compared with male patients, as reflected by higher HAM-A scores (*p* = 0.03, Cohen’s *d* = −0.81). They also reported greater emotional distress, bodily discomfort, and more severe sleep disturbances, as indicated by higher scores in the PDQ-39 emotional well-being domain (*p* < 0.001, Cohen’s *d* = −1.46), PDQ-39 bodily discomfort (*p* = 0.02, *r* = 0.38), PSQI total score (*p* = 0.03, Cohen’s *d* = −0.70), and sleep disturbances (*p* = 0.02, *r* = 0.37) (Table 1). Given the observed sex difference in bodily discomfort, we conducted a *post hoc* exploratory analysis of concomitant analgesic medication use, specifically non-steroidal anti-inflammatory drugs (NSAIDs), as a potential sex-related clinical variable. Fourteen out of 38 patients (36.8%) were receiving analgesicmedications; their use was numerically more frequent in women than in men (46.7% vs. 30.4%), although this difference did not reach statistical significance (Fisher’s exact test, *p* = 0.49). Analgesic medication use was not significantly associated with any caregiver burden dimension (all *p* > 0.40), but showed a trend toward higher PDQ-39 bodily discomfort scores (median 79.17 vs. 50.00, *p* = 0.075).

### 3.4 Correlation between patients and caregivers, stratified by patient sex

In the male patient subgroup, several significant correlations were observed between clinical outcomes and caregiver-related variables. IADL scale was strongly associated with greater caregiver burden (*r* = −0.83, *p* < 0.001, FDR corrected *p* < 0.001), increased time dedicated to assistance (*r* = −0.80, *p* < 0.001, FDR corrected *p* < 0.001), higher perceived disease progression (*r* = −0.74, *p* < 0.001, FDR corrected *p* < 0.001), and greater physical burden experienced by the caregiver (*r* = −0.64, *p* < 0.001, FDR corrected *p* = 0.004). Additionally, IADL scores were negatively correlated with the caregiver’s emotional burden (*r* = −0.44, *p* = 0.04, FDR corrected *p* = 0.11).

Similar patterns were found with the ADL scale. Specifically, lower ADL scores were significantly associated with higher caregiver burden (*r* = −0.69, *p* < 0.001, FDR corrected *p* = 0.002), longer caregiving time (*r* = −0.72, *p* < 0.001, FDR corrected *p* < 0.001), perception of disease progression (*r* = −0.60, *p* = 0.002, FDR corrected *p* < 0.001). A non-significant association was observed with physical burden (*r* = 0.42, *p* = 0.05, FDR-corrected *p* = 0.11), along with a non-significant trend for social burden (*r* = −0.39, *p* = 0.06, FDR-corrected *p* = 0.13).

Regarding emotional and psychological symptoms, anxiety levels (HAM-A) in male patients were positively correlated with perception of disease progression (*r* = 0.46, *p* = 0.03, FDR corrected *p* = 0.09), and social burden (*r* = 0.43, *p* = 0.04, FDR corrected *p* = 0.11). Trend was observed with caregiver burden (*r* = 0.42, *p* = 0.05, FDR-corrected *p* = 0.11).

Finally, better cognitive performance as measured by MMSE showed a non-significant trend toward lower time of caregiver assistance (*r* = −0.50, *p* = 0.01, FDR corrected *p* = 0.06), suggesting that greater cognitive impairment in patients may increase caregiver involvement.

In contrast, within the female patient subgroup, lower ADL scores were associated with greater time dedicated to assistance by the caregiver at the uncorrected level (*r* = −0.61, *p* = 0.02) but this association did not survive FDR correction (FDR correction *p* = 0.48). A similar non-significant trend was observed for caregiver burden (*r* = −0.51; *p* = 0.05).

### 3.5 Between and within group analysis in the caregiver group

After stratifying the caregiver sample by sex, none of the burden dimensions differed significantly between male and female caregivers (Table 2).

Within the subgroup of male caregivers, Spearman analysis revealed several moderate to large associations between patients’ functional independence to caregiving indices, although none survived FDR correction. Lower scores on patients’ ADL tended to coincide with a greater overall burden (*r* = −0.66, *p* = 0.010; FDR corrected *p* = 0.10), more time devoted to assistance (*r* = −0.69, *p* = 0.001; FDR corrected *p* = 0.10) and a more perceived disease progression by the caregiver (*r* = −0.54, *p* = 0.040; FDR corrected *p* = 0.24). A comparable, though slightly weaker, pattern was observed for IADL with caregivers’ perception of progression (*r* = −0.55, *p* = 0.030; FDR corrected *p* = 0.24). Conversely, better patient cognitive performance on the MMSE showed a positive trend with perceived progression (*r* = 0.58, *p* = 0.020; FDR corrected *p* = 0.24). No meaningful relationships emerged between patients’ affective symptoms (HAM-D, HAM-A) and any caregiver dimension.

Within the subgroup of female caregivers a coherent pattern of strong, inverse associations emerged between patients’ functional independence and several burden indices, and all of these remained significant after FDR correction. IADL scores were linked to a higher global burden (*r* = −0.73, *p* < 0.001, FDR corrected *p* < 0.001), more hours of daily assistance (*r* = −0.76, *p* < 0.001, FDR corrected *p* < 0.001), a higher perceived disease progression (*r* = −0.67, *p* < 0.001, FDR corrected *p* = 0.004) and greater physical burden (*r* = −0.57, *p* = 0.004, FDR corrected *p* = 0.02). A very similar profile was observed for basic ADL: poorer ADL performance correlated with global burden (*r* = −0.60, *p* = 0.002, FDR corrected *p* = 0.02), time spent caregiving (*r* = −0.74, *p* < 0.001, FDR corrected <0.001) and perceived progression of the illness (*r* = −0.55, *p* = 0.006, FDR corrected *p* = 0.03). No association involving the social or emotional burden subscales survived FDR adjustment, and patients’ affective (HAM-D, HAM-A) or cognitive (MMSE) scores showed no significant relationships with any caregiver dimension. These findings indicate that, for female caregivers, the patient’s loss of functional autonomy is the primary driver of objective and subjective burden (Table 2).

## 4 Discussion

This study explores the multidimensional burden experienced by caregivers of subjects with advanced PD, particularly those at Hoehn and Yahr stages IV–V, with a focus on the complex interplay between patients’ clinical characteristics and caregiver strain. It also addresses a critical yet often underexplored dimension in the caregiving literature: the role of sex in shaping both the clinical manifestation of patient symptoms and the caregiver’s experience of burden.

### 4.1 Functional decline as a central driver

The study found that decreased patient autonomy is strongly correlated with increased caregiver burden across emotional, physical, and social domains consistent with previous findings reported in literature (Genç et al., 2019; Rosqvist et al., 2022). As patients lose independence, caregivers are compelled to devote more hours to personal care, household tasks, and constant supervision. This not only generates significant physical fatigue but also emotional distress stemming from witnessing the deterioration of a loved one (Mosley et al., 2017). The unpredictable nature of PD, marked by motor fluctuations, freezing episodes, and hallucinations, further contributes to uncertainty and emotional strain (Chaudhuri et al., 2006; Martinez-Martin et al., 2023).

Notably, caregivers of patients at advanced stages are particularly vulnerable to chronic stress, burnout, and social isolation (Miyashita et al., 2009; Tessitore et al., 2018). The progressive nature of caregiving can also compromise caregivers’ personal identity and emotional (Mosley et al., 2017).

### 4.2 Quality of life and neuropsychiatric burden

The association between worse patient-reported QoL (especially regarding mobility, bodily discomfort, and ADL performance) and higher caregiver burden further supports the notion that QoL measures in PD are crucial for capturing the daily reality of caregiving demands. Although analgesic use was numerically more frequent in women, it was not significantly associated with caregiver burden in this cohort, suggesting that perceived pain may not directly translate into increased caregiver strain.

Recent studies confirm that advanced PD patients with severe QoL deterioration frequently require greater assistance, leading to higher stress and even burnout among informal caregivers (Tessitore et al., 2018; Rosqvist et al., 2022).

Interestingly, higher patient-reported anxiety was significantly more prevalent among female patients and was associated with a worse emotional well-being domain. This pattern resonates with other research suggesting that women with PD tend to report higher affective symptom severity than men, which can influence their support needs and complicate caregiving (Cerri et al., 2019). Fare clic o toccare qui per immettere il testo. However, although affective symptoms such as anxiety and depression may exacerbate caregiver stress, our findings suggest that they did not emerge as the predominant drivers of burden in this cohort. Rather, caregiver strain was more consistently related to patients’ functional dependence and, to a lesser extent, cognitive decline. Importantly, this should not be interpreted as minimizing the broader neuropsychiatric burden in advanced PD, since dementia and psychosis are closely intertwined with loss of autonomy and have been shown to substantially increase caregiving demands In line with this interpretation, cognitive impairment emerged as a key independent predictor of caregiver time and burden:

lower MMSE scores correlated with more caregiver involvement, reinforcing the relationship between cognitive decline and caregiving complexity.

These results echo prior research showing that both mild cognitive impairment and PD dementia dramatically heighten caregiver stress, disability, and overall burden (Santos-García and de la Fuente-Fernández, 2015; Karlstedt et al., 2020).

In this sense, affective, cognitive, and functional dimensions likely interact in shaping caregiver burden, although in our cohort functional dependence and cognitive decline appeared to play the most prominent role.

### 4.3 Sex-sensitive patterns

The stratified analysis revealed noteworthy sex-based variations in both patient symptomatology and caregiver burden dynamics, echoing and expanding upon findings in the broader literature. While female patients reported significantly higher levels of anxiety, emotional distress, and sleep-related difficulties, these symptoms did not translate into equally strong caregiver burden correlations. In contrast, caregivers of male patients reported significantly stronger associations between patients’ functional decline (both ADL and IADL scores) and perceived burden, caregiving time, and emotional strain.

These discrepancies may reflect both biological and sociocultural influences. From a clinical perspective, male PD patients may be more likely to exhibit externalizing symptoms such as impulsivity, disinhibition, or behavioral dysregulation, which can be particularly distressing for caregivers (Georgiev et al., 2017). Conversely, while female patients may experience higher internalizing symptoms such as anxiety and sleep disturbance, these may be less disruptive to daily caregiving routines, especially when the caregiver is emotionally attuned to such expressions (Mahale et al., 2021).

On the caregiver side, sex roles likely influence how burden is experienced and reported. Female caregivers, who showed stronger correlations between patient autonomy and global burden, may assume more comprehensive caregiving roles, including physical care, emotional support, and medical management (Mosley et al., 2017; Carpinelli et al., 2023). Despite these heavy responsibilities, female caregivers may underreport emotional distress, as societal gender norms often frame caregiving as a natural and expected extension of women’s family role, potentially leading to an underrecognition of their psychological burden and affecting the interpretation of caregiver burden questionnaires (White and Palmieri, 2024). Caregivers may experience substantial distress when caregiving involves sustained emotional adaptation, complex care coordination, and management of behavioral or neuropsychiatric symptoms. Although some studies suggest sex-related differences in caregiver burden in PD (Grün et al., 2016; Balash et al., 2019), others have not confirmed consistent differences between male and female caregivers, and the underlying mechanisms remain unclear (Lee et al., 2019; Klietz et al., 2021).

Interestingly, in the female patient subgroup, the most consistent association was observed between ADL decline and caregiving time. However, this finding should be interpreted with caution, as the smaller size of this subgroup may have reduced the statistical power to detect additional associations with other burden dimensions. Therefore, subgroup-specific patterns in female patients require confirmation in larger cohorts. Taken together, these findings support the idea that caregiver burden in PD is not monolithic, but shaped by a dynamic interplay of patient and caregiver sex, symptom profile, and psychosocial context. This underscores the need for sex-sensitive assessment tools and interventions that account for these nuanced interactions. More broadly, the literature on sex differences in PD caregiving remains mixed, but larger studies suggest that patient sex may meaningfully influence caregiving patterns and perceived strain. In a large international cohort of 7,209 individuals with PD from the Parkinson’s Outcomes Project, men were more likely than women to have an informal caregiver, and caregivers of male patients reported significantly greater strain than caregivers of female patients, even after adjustment for age, disease stage, comorbidities, cognition, mobility, and health-related QoL (Dahodwala et al., 2018). These findings are consistent with evidence that caring for a male patient may be associated with greater caregiver strain, particularly among female spouses (Greenwell et al., 2015), and with observations from the international late-stage cohort of which the current sample is a part (Kalampokini et al., 2022). However, smaller studies have not always confirmed this association; for example, Klietz et al. (2021) in a cohort of 84 patient-caregiver dyads, found no significant sex differences in overall caregiver burden.

Although our study did not directly assess social support or access to healthcare services, these contextual factors are likely to influence caregiver burden and may partly contribute to the sex-sensitive patterns observed in our cohort. Differences in family support, access to community resources, and availability of multidisciplinary care may shape how caregiving demands are experienced and managed. These considerations reinforce the importance of multidisciplinary and sex-sensitive care pathways in advanced PD, integrating neurological care with psychological support, rehabilitation, caregiver education, social services, and respite interventions (Aye et al., 2020).

These findings suggest that caregiver support in advanced PD should be tailored to the specific needs of each caregiver–patient dyad. A sex-sensitive perspective may still be useful in identifying different burden patterns, but interventions should remain individualized and may include psychological support, stress-management strategies, practical training, respite care, and support in managing behavioral and neuropsychiatric symptoms. Future studies should explore caregiver–patient dyads through an intersectional lens that includes sex, relationship type (spouse vs. child), and caregiving context (cohabiting vs. separate households), in order to better understand the mechanisms underlying differential burden trajectories.

### 4.4 Caregiver interventions in Parkinson’s disease

Despite strong recommendations to support caregivers in chronic illness, interventions specifically targeting caregivers of individuals with PD, particularly in advanced stages, remain scarce. One of the best-known examples is the Patient Education Program Parkinson (PEPP) a structured educational intervention developed to support patients and caregivers by improving disease-related knowledge, coping skills, and QoL, as a complement to medical treatment, and based on the biopsychosocial model of the International Classification of Functioning, Disability and Health (ICF) (Macht et al., 2007). However, most existing programs, including PEPP, have historically focused on patient-centered outcomes rather than addressing caregivers’ long-term psychological needs. Although PEPP demonstrated short-term reductions in caregiver burden (A’Campo et al., 2010a, 2010b), improvements in QoL were limited, and benefits tended to diminish within 6 months. Notably, caregivers consistently valued stress management and peer support modules, with response rates approaching 80%.

Other strategies, including self-management approaches and multicomponent caregiver training programs, remain promising but are rarely implemented in standard care pathways (Mohd Amin et al., 2024). A structured eight-session educational intervention for persons with PD and their caregivers, incorporating modules on pleasant activity scheduling, stress management, communication skills, and burden-reduction techniques, has also shown effectiveness in reducing caregiver burden compared to both baseline and usual care, with more pronounced effects among caregivers of cognitively intact patients (Simons et al., 2006; A’Campo et al., 2010a, 2010b, 2012). Moreover, a cognitive behavioral therapy (CBT) program consisting of 12–14 individualized sessions for caregivers reporting high emotional stress demonstrated significant reductions in burden, with sustained effects at six-month follow-up (Secker, 2005). Qualitative evidence further supports the potential benefits of educational interventions and opportunities for peer fellowship among caregivers (Schrag et al., 2004; Roland et al., 2010; McLaughlin et al., 2011; Tan et al., 2012), although the roles of peer support and mindfulness-based approaches have yet to be rigorously investigated (Shah et al., 2015; Cash et al., 2016).

### 4.5 Limitations, strengths, and future directions

This study highlights several critical points for both clinical practice and future research. One of the main limitations is the relatively small sample size, which may restrict the generalizability and statistical power of the findings. Further research involving larger and more diverse cohorts will be essential to confirm and expand upon these observations. Additionally, although the CBI is a validated multidimensional tool, it is not specifically designed for PD; future studies could benefit from incorporating PD-specific instruments such as the Zarit Burden Interview or the Parkinson’s Disease Caregiver Burden Scale (Carrilho et al., 2018) to achieve a more nuanced evaluation.

Another limitation is that caregiver-specific psychosocial variables, such as coping strategies, resilience, and overall emotional health, were not directly measured, despite their potential role in mediating perceived burden. Furthermore, non-motor symptoms were not systematically assessed using a dedicated comprehensive scale. In addition, data on psychotic symptoms in patients, such as hallucinations or delusions, were not systematically collected. This represents a further limitation, as psychosis is common in advanced PD and may substantially contribute to caregiver burden and distress. While some domains such as cognition, anxiety, depression, sleep, and QoL were examined, other relevant non-motor symptoms may have been under-reported and under-recognized. This is an important limitation, as non-motor symptoms are highly prevalent in advanced PD, may show sex-related differences, and may affect caregiver burden as perceived by both patients and caregivers (Cattaneo et al., 2025). Likewise, Unified Parkinson’s Disease Rating Scale (UPDRS) data were not systematically available in the present cohort; therefore, we could not assess the potential relationship between motor symptom severity and caregiver burden. Furthermore, given the exploratory nature of the study and the relatively limited sample size, we deliberately refrained from performing multivariate analyses such as multiple regression or structural modeling. Such approaches require larger cohorts to ensure statistical robustness and avoid overfitting. Instead, we focused on non-parametric bivariate correlations and stratified comparisons, which are more appropriate and statistically reliable given the characteristics of the sample and study design.

From a clinical standpoint, these results emphasize the need for regular functional and cognitive assessments in patients with advanced PD, not only to optimize patient care but also to proactively identify caregivers who may be at heightened risk of stress or burnout. Integrating caregiver-focused supports, such as structured psychoeducation, telehealth consultations, peer support networks, and access to respite care, into multidisciplinary care pathways is crucial. Importantly, these interventions should be designed within a sex-sensitive framework, recognizing the different ways in which men and women may experience and respond to caregiving demands.

Future research should consider adopting dyadic, preferably longitudinal, study designs to capture the dynamic interplay between patients and their caregivers over time. Developing tailored interventions that combine psychological counseling, training in symptom management, digital resources, and periodic booster sessions could help sustain caregiver well-being and resilience, ultimately improving outcomes for both caregivers and patients in advanced stages of Parkinson’s disease.

## 5 Conclusion

In conclusion, this study highlights the pivotal impact of reduced patient autonomy, cognitive impairment, and diminished QoL on caregiver burden in advanced stages of PD. Our results confirm that as functional independence deteriorates, caregivers face escalating demands across physical, emotional, and social domains, often leading to chronic stress and burnout. Importantly, sex-stratified analyses revealed that sex influences how patients’ symptoms are experienced and how caregiving responsibilities are managed, emphasizing the need for sex-sensitive support strategies.

From a clinical perspective, regular monitoring of both patients’ functional status and caregivers’ well-being is crucial to identify those at greatest risk for psychological distress and health decline. Integrating caregiver-focused components, such as psychoeducation, peer support, telehealth solutions, and respite programs, within multidisciplinary care models may help address the evolving needs of caregivers, especially in advanced PD.

## Data Availability

The raw data supporting the conclusions of this article will be made available by the authors, without undue reservation.
